# Counterintuitive relationship between the triglyceride glucose index and diabetic foot in diabetes patients: A cross-sectional study

**DOI:** 10.1371/journal.pone.0293872

**Published:** 2023-11-03

**Authors:** Zhaoping Li, Minghao Zhang, Lulu Han, Lili Fu, Yixia Wu, Haiyan Chen, Li Feng

**Affiliations:** 1 Department of Clinical Nutrition, Shandong Provincial Hospital Affiliated to Shandong First Medical University, Taian, Shandong, China; 2 Department of Geriatric Endocrinology, Shandong Provincial Hospital Affiliated to Shandong First Medical University, Taian, Shandong, China; Muhimbili University of Health and Allied Sciences School of Medicine, UNITED REPUBLIC OF TANZANIA

## Abstract

**Background:**

Research has shown that insulin resistance (IR) is a known risk factor for diabetic foot (DF), and the triglyceride-glucose (TyG) index is a reliable and simple indicator of IR. However, less is known about the relationship between the TyG and the risk of DF. Here, we investigated the association between the TyG index and the prevalence of DF.

**Methods:**

The eligible records from the Departments of Endocrinology of Shandong Provincial Hospital Affiliated to Shandong First Medical University were screened (from December 1, 2012, to December 31, 2021), and a total of 8866 patients were enrolled. The TyG index was calculated as ln[(fasting triglycerides (mg/dL)×fasting glucose (mg/dL)/2)]. The continuous variables between the DF and the non-DF groups were compared by Student’s t test or the Mann-Whitney U test, and categorical variables were compared by the chi-square test. Receiver operating characteristic curve (ROC) analysis was carried out to estimate the predictive value of the TyG index for DF. Logistic regression models were used to evaluate the associations between the quartiles of the TyG index and the risk of DF. Subgroup and sensitivity analyses were conducted.

**Results:**

The TyG index was significantly lower in the DF group than in the no-DF group. The logistic regression revealed that an increased TyG index was associated with a lower risk of DF after adjusting for potential confounders. In addition, an ROC analysis indicated the discriminatory ability of the TyG index in DF presence with an area under the curve (AUC) of 0.661 (95% CI 0.642–0.680, P < 0.001). Subgroup and sensitivity analysis also supported these robust results.

**Conclusions:**

The TyG index was inversely and dose-dependently associated with the risk of DF in diabetes patients, indicating that elevated TyG index was a protective factor for DF. Future studies are therefore warranted to confirm our finding and to explore the detailed pathological mechanism involved in this process.

## Introduction

During recent decades, the prevalence of diabetes mellitus (DM) is on the rise and was estimated at 10.5% (537 million) worldwide in 2021 based on the 10th edition of the IDF Diabetes Atlas 2021 [1]. The number of people diagnosed with DM is estimated to be 643 million by 2030 and 783 million by 2045 globally [2]. In addition, a concerning finding showed that 8.5% of the world population has a diagnosis of diabetes [3]. A 2018 report shows that 23.4 million citizens (9.72%) have diagnosed diabetes and 81.6 million citizens (33.9%) have prediabetes in the US [4]. Diabetic foot is the most serious complication of diabetes [5]. The global prevalence of diabetic foot (DF) is nearly 6.3% [6], which leads to the impaired quality of life and even reduced life expectancy. In a previous study [7], it was shown that approximately 25% of diabetes patients suffered from foot ulcers in their lifetime. Furthermore, the increasing disease-related cost of DF has become a serious public health problem. Ronald L. Horswell et al. [8] reported that a 12-month staged management of foot ulcers resulted in lower foot-related inpatient days (0.91 d per person vs. 3.97 d per person) and inpatient charges ($1321 per person vs. $5411 per person). Therefore, early identification of risk factors and integrated management are important and necessary to treat and prevent DF.

In terms of pathophysiology, multifactorial factors result in DF, including peripheral vascular damage caused by chronic insulin resistance (IR), sensation loss due to peripheral neuropathy, poor glycemic control, diabetes duration, joint deformity, smoking and some other potential factors [9–11]. Among these factors, hyperglycemia and IR might contribute to vascular trauma in diabetes [12], and this peripheral vascular damage plays a huge role in the occurrence and development of DF [9].

Hyperinsulinemic euglycemic clamp was reported to accurately assess the IR in peripheral tissues [13], but the complex operation and high cost limited its extensive use [14]. The homeostasis model assessment of insulin resistance (HOMA-IR) is a validated marker of IR, but it is difficult to truly reflect the dynamic process of insulin secretion by inferring the dynamic function of pancreatic islet β cells from fasting steady-state data [15]. Novel insulin sensitivity indices derived from oral glucose tolerance tests, such as the insulinogenic index (IGI), Stumvoll index and Matsuda index [16], can be used for the evaluation of individual insulin sensitivity and the secretion function of pancreatic islet β cells [17]. The cumbersome calculation limited their clinical practice. The triglyceride-glucose (TyG) index has been reported to be a reliable and simple indicator of IR [18], which was calculated based on fasting blood glucose (FBG, mg/dL) and plasma total triglycerides (TG, mg/dL) [19]. However, evidence of the relationship between blood glucose and lipids and the occurrence of DF is quite limited.

Since the role of FBG and TG in IR has been verified [20, 21], TyG index, as a combined product of FBG and TG, might show a better predictive performance to identify IR in DF. No previous studies have focused on the relationship between the TyG index and DF presence in patients with diabetes. Therefore, in the present study, we investigated the relationship between the TyG index and the risk of DF by using a large cross-sectional study based on inpatients with diabetes.

## Methods

### Data source and study population

This was a cross-sectional study, and all data used in the study were extracted from YiduCloud (Technology Co., Ltd. Jinan, China), which is a big data intelligent platform that integrates and converges massive electronic medical record data from multiple medical institutions in China. All the information on the platform was anonymous and had unique identified codes for privacy protection. The medical records of the Departments of Endocrinology of the Shandong Provincial Hospital Affiliated to Shandong First Medical University (Jinan, Shandong, China) from January 2012 to December 2021 were screened. Patients who were less than 18 years old, pregnant, or lacking weight, height, circulating triglycerides or glucose concentrations were excluded. Eventually, a total of 8866 hospitalized diabetes patients were enrolled (S1 Fig). All data from medical records were fully anonymized and there was no information that could identify individual participants, so the ethics committee waived the requirement for informed consent. The study was approved by the Ethics Committee of the Shandong Provincial Hospital Affiliated to Shandong First Medical University (SWYX: NO.2022-071).

### Data collection

The following clinical variables were extracted and analyzed: age, sex, duration of diabetes, height, weight, systolic blood pressure (SBP), diastolic blood pressure (DBP), drinking and smoking status. In addition, blood indices during hospitalization were also extracted: FBG (fasting blood glucose), FINS (fasting plasma insulin concentration), TC (total cholesterol), TG (triglyceride), HDL-C (high-density lipoprotein cholesterol), LDL-C (low-density lipoprotein cholesterol), APOA (apolipoprotein A), APOB (apolipoprotein B), ALB (serum albumin), PA (prealbumin), TP (total protein), GLO (globulin), HBG (hemoglobin) WBC (white blood cell), RBC (red blood cell), NEUT#, (numbers of neutrophils), PLT (blood platelets), ALT (glutamic-pyruvic transaminase), CREA (creatinine), URIC (uric acid), glycated hemoglobin (HbA1c%) and C-reactive protein (CRP).

### Outcome and covariates

Diagnosing diabetic foot ulcers was in line with the WHO criteria [22], as an ulcerative lesion of the foot (including the ankle) that was related to peripheral neuropathy, vascular disease and infection. Ulcerative foot injury was defined as a defect of the full-thickness skin that required more than 14 days for healing [23]. The TyG index was calculated as follows [24]: TyG = ln [(fasting triglycerides (mg/dL) ×fasting glucose (mg/dL)/2)].

HOMA-IR was used to estimated degree of IR. HOMA-IR = FPG (mmol/L) × FINS (μU/ml)/22.5 [25]. Pulse pressure (PP) was calculated by SBP minus DBP. Smoking was defined as self-reported smoking history or current smoking. Drinking was defined as self-reported drinking history or current drinking, regardless of frequency. BMI was calculated as the weight (kg) divided by height in meters squared (m^2^). Overweight was defined as BMI ≥24 kg/m^2^ [26]. Utilization of insulin and other oral hypoglycemic drugs, including insulin secretagogues, biguanides, glycosidase inhibitors, thiazolidinediones and DPP4 inhibitors were collected. Lipid-lowering agents including fenofibrate agents and statin drugs were also included as covariates.

### Statistical analysis

Continuous variables with a normal distribution are expressed as the mean±standard deviation; otherwise, they are expressed as the median and interquartile range. Categorical variables were expressed as percentages. The Shapiro‒Wilk test was used to assess the normal distribution of each continuous variable. The continuous variables between the DF and the non-DF groups were compared by unpaired Student’s t tests or Wilcoxon rank-sum tests, and categorical variables were compared by chi-square tests. To include as many cases as possible, a category for unknown was created due to the systematic missing data in TC, LDL-C, HDL-C, APOA/APOB, ALB, PA, GLO, HBG, WBC, RBC, NEUT#, PLT, ALT, CREA, URIC, HbA1c% and CRP. The TyG index was analyzed in quantiles. Quartiles were defined as the 25th, 50th, and 75th percentiles of the TyG index (quartile 1 [Q1] 6.18–8.66; quartile 2 [Q2] 8.66–9.16, quartile 3 [Q3] 9.16–9.69, quartile 4 [Q4] 9.69–12.94) with Q1 being the reference quartile. Multivariable logistic regression models were used to evaluate the associations between the TyG index and the risk of DF. Model 1 was unadjusted; Model 2 was adjusted for age and sex; Model 3 was adjusted for the variables in model 2 plus smoking and drinking; and Model 4 was adjusted for the variables in model 3 plus BMI, duration of diabetes, PP, TC, LDL-C, HDL-C, APOA/APOB, ALB, PA, GLO, HBG,PLT, WBC, RBC, NEUT^#^, ALT, CREA, URIC, HbA1c%, CRP, fenofibrate agents, statin drugs, insulin, insulin secretagogues, biguanides, glycosidase inhibitors, thiazolidinediones and DPP4 inhibitor. The restricted cubic spline model was used for the dose‒response analysis between TyG index and risk for DF presence. Furthermore, subgroup analyses stratified by sex (male, female), age (<60, ≥60) and overweight status (yes, no) were performed. Due to the small numbers of T1DM (Type 1 diabetes), stratified analyses based on different diabetes types were not able to be conducted. Therefore, a sensitivity analyse by excluding T1DM was conducted to assess the robustness of the results. A receiver-operating characteristic (ROC) analysis was used to estimate the predictive value of the TyG index for DF presence based on the value of the area under the ROC curve (AUC). All data were analyzed using Empower software (www. empowerstats.com; X&Y solutions, Inc., Boston MA) and R software 3.6.2. A P value of < 0.05 was considered to indicate statistical significance.

## Results

### The demographic and clinical characteristics of patients with and without DF

The characteristics of the included participants are summarized in Table 1. A total of 8866 participants were included in the analysis, including 5034 males and 3832 females, with a mean age of 55.27 (13.96) years and a mean diabetes duration of 9.33 (7.63) years. A total of 8.83% (783/8866) of patients were identified with DF. Compared with the non-DF group, DF patients were more likely to be male, current smokers and drinkers, elderly, hypoalbuminemic, have a longer duration of diabetes, and have a higher SBP and PP (all P < 0.001). However, patients with DF had significantly lower concentrations of FBG, TC, TG, LDL and HDL, accompanied by a lower TyG index and lower BMI (all P < 0.001).

**Table 1:** Characteristics of the participants.

	total	non-DF group	DF group	P value
Variables	n = 8866	n = 8083	n = 783	
Age, years	55.27±13.96	54.42±13.93	64.08±10.96	<0.001
Male, n (%)	5034 (56.78)	4530 (56.04)	504 (64.37)	<0.001
Smoking, n (%)	3208 (36.18)	2844 (35.18)	364 (46.49)	<0.001
Drinking, n (%)	3567 (40.23)	3209 (39.70)	358 (45.72)	<0.001
Duration of diabetes, year	9.33±7.63	8.91±7.46	13.62±8.11	<0.001
BMI, kg/m^2^	25.66±4.05	25.75±4.08	24.70±3.56	<0.001
FBG, mmol/L	9.29±4.61	9.42±4.65	7.91±3.91	<0.001
TG, mmol/L	1.86±1.86	1.91±1.93	1.31±0.78	<0.001
TC, mmol/L	5.02±1.42	5.07±1.42	4.50±1.22	<0.001
LDL-C, mmol/L	3.11±1.00	3.14±1.00	2.79±0.91	<0.001
HDL-C, mmol/L	1.17±0.33	1.17±0.33	1.11±0.31	<0.001
APOA, mmol/L	1.09±0.21	1.10±0.21	0.97±0.22	<0.001
APOB, mmol/L	1.03±0.34	1.04±0.34	0.95±0.33	<0.001
TyG index	9.16 (1.03)	9.20 (1.03)	8.78 (0.85)	<0.001
SBP, mmHg	136.58±20.87	136.10±20.59	141.56±23.00	<0.001
DBP, mmHg	82.25±12.54	82.51±12.50	79.49±12.59	<0.001
PP, mmHg	54.34±16.66	53.59±16.34	62.07±17.92	<0.001
HbA1c%>6.5%, n (%)	6227 (70.23)	5639 (69.76)	588 (75.10)	<0.001
ALB<40 g/L, n (%)	3489 (39.35)	2956 (36.57)	533 (68.07)	<0.001
Fenofibrate, n (%)	739 (8.34)	731 (9.04)	8 (1.02)	<0.001
Statin drugs, n (%)	4665 (52.62)	4252 (52.60)	413 (52.75)	0.940
Insulin, n (%)	5520 (62.26)	4926 (60.94)	594 (75.86)	<0.001
Insulin secretagogues, n (%)	1891 (21.33)	1780 (22.02)	111 (14.18)	<0.001
Biguanides, n (%)	4263 (48.08)	3946 (48.82)	317 (40.49)	<0.001
Glycosidase inhibitors, n (%)	4967 (56.02)	4497 (55.64)	470 (60.03)	0.018
Thiazolidinediones, n(%)	90 (1.02)	81 (1.00)	9 (1.15)	0.695
DPP4 inhibitor, n (%)	2515 (28.37)	2288 (28.31)	227 (28.99)	0.685

Data are mean (SD), medians (interquartile ranges) or percentage. The P value was estimated using the chi-square test for proportions, unpaired Student’s t tests for means or the Wilcoxon rank-sum test for medians. BMI, body mass index; FBG, fasting blood glucose; TC, total cholesterol; TG, triglyceride; LDL-C, low-density lipoprotein cholesterol; HDL-C, high-density lipoprotein cholesterol; APOA, apolipoprotein A; APOB, aolipoprotein B; TyG index, triglyceride glucose index; SBP, systolic blood pressure; DBP, diastolic blood pressure; PP, pulse pressure; ALB, serum albumin; DPP4 inhibitor, dipeptidyl peptidase 4 inhibitor.

### Associations of the TyG index and DF

A logistic regression model was conducted to assess the association between the quartiles of the TyG index and the prevalence of DF (Table 2). Quartile 2, Quartile 3 and Quartile 4 all had a lower risk of DF than Group Q1 in the single-factor analysis. The result was still analogous in Model 2 (adjusted by age and sex), Model 3 (adjusted by smoking and drinking & Model 2). After adjustment for age, sex, smoking, drinking, BMI, duration of diabetes, PP, TC, LDL-C, HDL-C, APOA/APOB, ALB, PA, GLO, HBG, PLT, WBC, RBC, NEUT#, ALT, CREA, URIC, HbA1c%, CRP, fenofibrate agents, statin drugs, insulin, insulin secretagogues, biguanides, glycosidase inhibitors, thiazolidinediones and DPP4 inhibitor in Model 4, the TyG index was still inversely associated with the risk of DF in the Q2 group (OR 0.75; 95% CI 0.60, 0.93), Q3 group (OR 0.58; 95% CI 0.45, 0.75), and Q4 group (OR 0.40; 0.28, 0.55) respectively. The OR value decreased with the increase in the quantiles of the TyG index (P for trend < 0.001).

**Table 2:** The association between TyG index and DF.

TyG index		continuous	TyG index, quartile				P_trend_
			Q1	Q2	Q3	Q4	
**Odds ratio (95% CI)**	Model 1	0.48 (0.43, 0.53)	Ref	0.63 (0.53, 0.76)	0.39 (0.32, 0.48)	0.19 (0.15, 0.25)	<0.001
	Model 2	0.56 (0.50, 0.62)	Ref	0.69 (0.57, 0.83)	0.47 (0.38, 0.58)	0.29 (0.22, 0.38)	<0.001
	Model 3	0.55 (0.50, 0.62)	Ref	0.69 (0.57, 0.83)	0.46 (0.37, 0.57)	0.28 (0.22, 0.37)	<0.001
	Model 4	0.68 (0.59, 0.79)	Ref	0.75 (0.60, 0.93)	0.58 (0.45, 0.75)	0.40 (0.28, 0.55)	<0.001

Model 1: unadjusted

Model 2: adjusted for age and sex

Model 3: further adjusted for smoking and drinking

Model 4: further adjusted for body mass index, duration of diabetes, pulse pressure, total cholesterol, LDL cholesterol, HDL cholesterol, APOA/APOB, total serum albumin, prealbumin, globulin, hemoglobin, platelets, white blood cells, red blood cells, numbers of neutrophils, glutamic-pyruvic transaminase, creatinine, uric acid, glycosylated hemoglobin, C reactive protein, fenofibrate agents, statin drugs, insulin, insulin secretagogues, biguanides, glycosidase inhibitors, thiazolidinediones and DPP4 inhibitors.

The quartile cutoff values of the TyG index were 8.66, 9.16 and 9.69.

**Fig 1:**
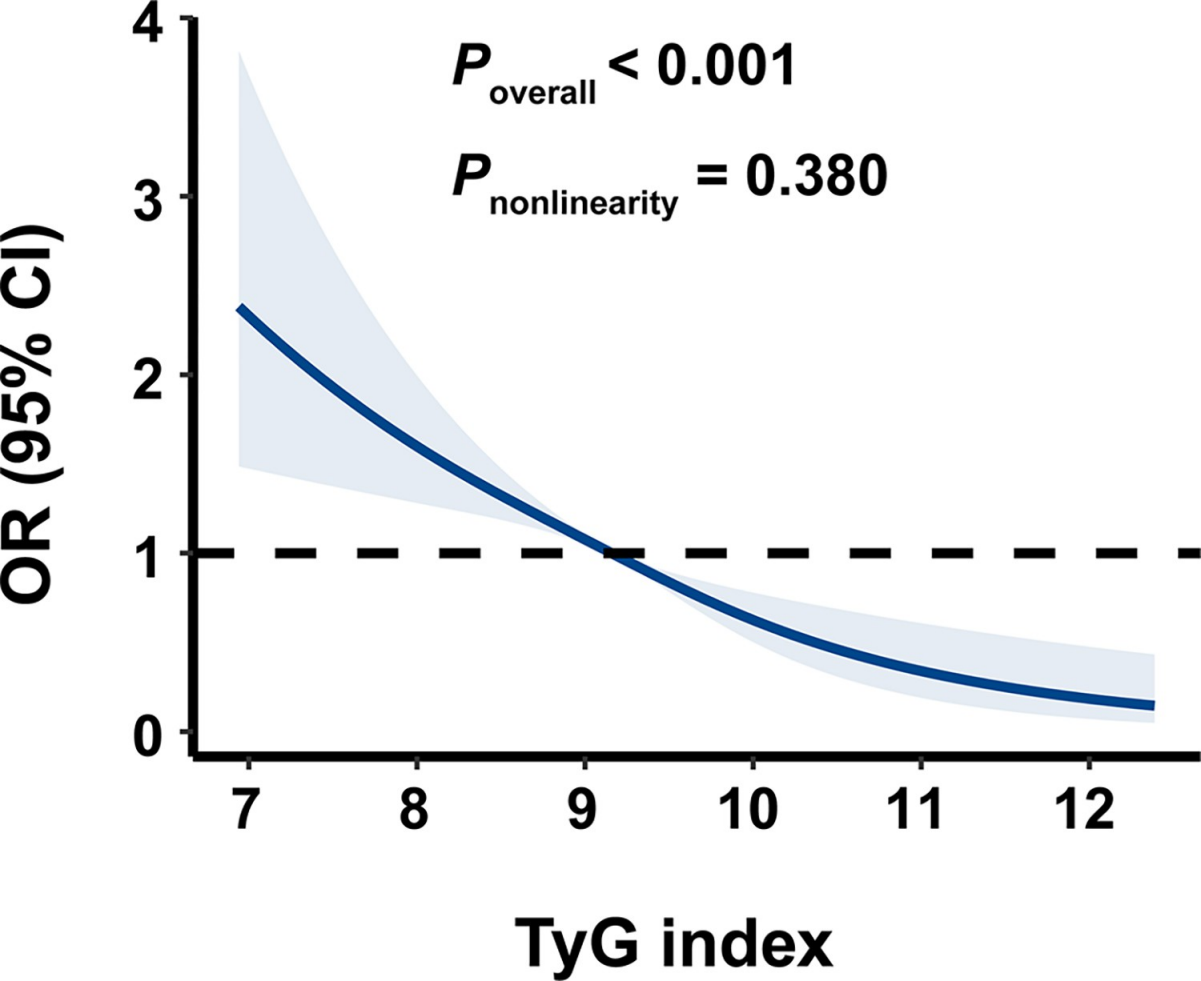
Dose-response association between the TyG index and the risk of DF. TyG, triglyceride glucose index; DF, diabetic foot.

As seen in Fig 1, the restricted cubic spline model showed a nearly linear relationship between the TyG index and the risk of DF after adjustment for multiple potential covariates in Model 4 (P for overall < 0.001, P for nonlinear = 0.380).

**Fig 2:**
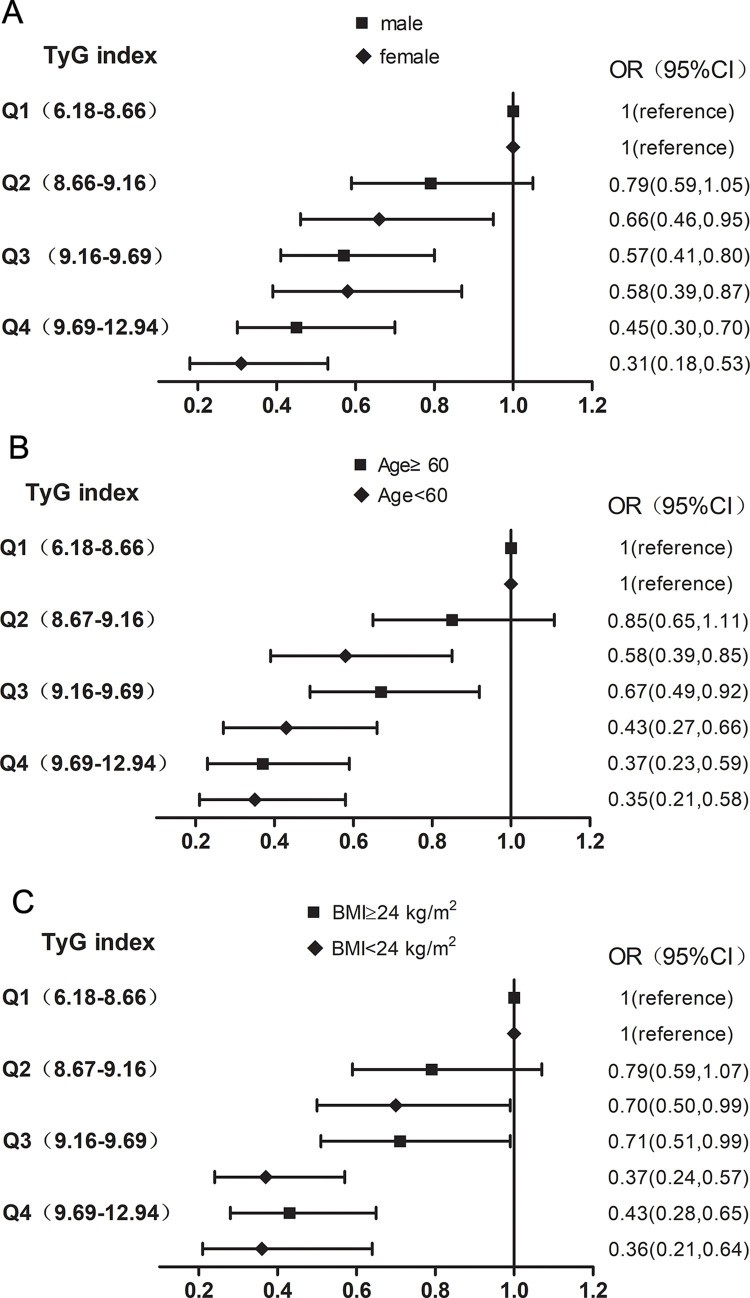
Subgroup analysis of odds ratios and 95% CIs of the TyG index with DF in diabetic patients. (A) at different gender, (B) at different age and (C) at different body mass index. TyG, triglyceride glucose index; DF, diabetic foot.

Then, subgroup analysis was conducted to further assess the robustness of the relationship between the TyG index and the risk of DF. All analyses were adjusted for age, sex, smoking, drinking, BMI, duration of diabetes, PP, TC, LDL-C, HDL-C, APOA/APOB, ALB, PA, GLO, HBG, PLT, WBC, RBC, NEUT#, ALT, CREA, URIC, HbA1c%, CRP, fenofibrate agents, statin drugs, insulin, insulin secretagogues, biguanides, glycosidase inhibitors, thiazolidinediones and DPP4 inhibitor except for the stratified variables. As shown in Fig 2 and S1 Table, the result was robust in the sense that subgroup analysis demonstrated the effect was independent of age, sex, and overweight status (all P for interaction>0.05).

To exclude the influence of different types of diabetes on this relationship, a sensitivity analyse was conducted by excluding T1DM. The association between the TyG index and the risk of DF was also stable (S2 Table).

To verify that the TyG index could reflect IR, a correlation analysis between the TyG index and HOMA-IR was conducted. Because of the large number of random missing data for fasting insulin levels, we conducted a sensitivity analyse by excluding subjects with missing data. The linear correlation of the TyG index with HOMA-IR was modest (Spearman rank correlation coefficient ρ = 0.477, P < 0.001). In addition, as shown in S3 Table, the TyG index was still inversely associated with the risk of DF in the Q2 group (OR 0.73; 95% CI 0.52, 1.03), Q3 group (OR 0.65; 95% CI 0.43, 0.97), and Q4 group (OR 0.38; 0.22, 0.63) in participants in this subgroup (P for trend < 0.001). However, HOMA-IR was not associated with the risk of DF in the Q2 group (OR 1.06; 95% CI 0.75, 1.51), Q3 group (OR 0.73; 95% CI 0.48, 1.11), or Q4 group (OR 0.91; 0.58, 1.42) after adjusting for covariates in Model 4 (P for trend = 0.3293).

### ROC analysis for the identification of patients with a risk of DF

**Fig 3:**
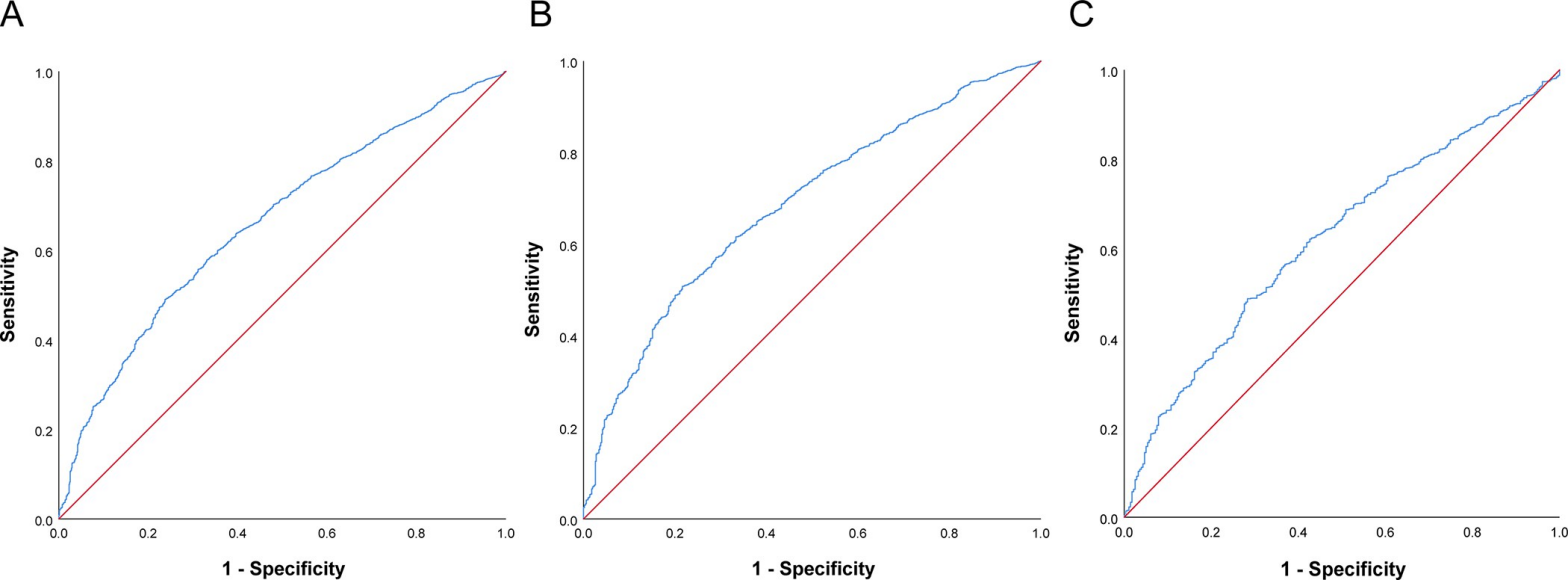
Receiver-operating characteristic analysis for identification of patients with a risk of DF. (A) overall population; (B) male; (C) female. DF, diabetic foot.

Receiver operating characteristic (ROC) curve analysis was conducted to evaluate the performance of the TyG index for identifying patients with a risk of DF. As shown in Fig 3, the area under the curve (AUC) of the TyG index was 0.661 (95% CI 0.642–0.680, P < 0.001) in the whole population, with an AUC of 0.686 (95% CI 0.663–0.709, P < 0.001) in males and 0.621 (95% CI 0.589–0.653, P < 0.000) in females. There was a higher AUC of the TyG index in men than that in women (δ_AUC_ = 0.065, 95% CI 0.026–0.104, P *=* 0.001). A sensitivity analyse excluding T1DM showed a similar result (S2 Fig).

## Discussion

In this large-scale study, we assessed the relationship between the TyG index and the risk of DF in diabetes patients, and an inverse relationship was identified. The association was consistent when stratified by age, sex and BMI. To our knowledge, no published reports have focused on the effects of the TyG index on DF presence. Additionally, the TyG index slightly improved the identification ability of DF presence in diabetes patients. Given that elevated TyG index was a protective factor for DF, keeping the TyG index at a relatively high level may be beneficial; however it should be necessary to state that the current results do not in any way suggest abandoning hypoglycemic and lipid-lowering therapy.

Generally, the TyG index is considered as a widely available and reliable surrogate marker for the early identification of IR [27], which is closely related to diabetic complications [18]. However, literature on the association between the TyG index and diabetic complications is inconsistent. Previous report revealed that the TyG index was a predictor for the incidence of type 2 diabetes [28] and an elevated TyG index was a risk factor for diabetic nephropathy [29] and chronic kidney disease (CKD) progression in T2DM [30]. L. Liu et al. [31] reported that the TyG index was independently associated with microalbuminuria in patients with T2DM but had no obvious correlation with the estimated glomerular filtration rate. Another study based on hospitalized patients from Pan et al. [32] noted that the TyG index was significantly related to microalbuminuria and ABI but not chronic kidney disease (CKD), diabetic retinopathy (DR), or brachial-ankle pulse wave velocity (ba-PWV). A U-shape relationship between the TyG index and the risk of diabetic retinopathy (DR) based on the National Health and Nutrition Examination Survey (NHANES) was reported by Zhou et al. [33]. Counterintuitively, a negative relationship between the TyG index and the risk of DF in diabetes patients was reported in our study. In short, the TyG index might be a protective factor against DF presence. This finding was supported by a nested case-control study [34] on the TyG index and the risk of DR, which reported that the TyG index might be a protective factor for DR and vision-threatening DR. In that study, the DR group had a lower TyG index, and logistic regression showed a dose‒response relationship between the TyG index and the presence of DR after adjusting for age, sex, duration of diabetes, use of hypoglycemic drugs, heart rate, SBP, PP, height, weight, BMI, HbA1c and TC [34]. The differences in study design and considerable heterogeneity of the participants are need to be taken into account to explain these divergent results. However, our results need to be interpreted with caution because some potential confounding factors, such as walking habits, joint deformities and dietary factors, were not considered.

In this study, a modest linear correlation between the TyG index and HOMA-IR was revealed, which verified that the TyG index was a reliable indicator of IR. However, the evaluation of insulin sensitivity itself had limitations, and the dynamic insulin sensitivity was not evaluated. Although our result was unexpected, we had reasons to believe that the TyG index was an affordable and easily utilized parameter of IR in clinical practice and could be expected to become a potential indicator for DF in diabetes patients. Additionally, the TyG index derived from FBG and triglycerides represented the joint effects of serum glucose and lipids. Further mediation and interaction analyses were wanted to clarify the effect of the TyG index on the risk of DF.

Both hyperglycemia and hypoglycemia can be associated with poor clinical outcomes [35]. Peled S et al. [36] reported that severe hypoglycemia and high glycemic variability were significantly associated with amputations in DF patients. We speculate that a lower TyG index of diabetes patients may be a marker of hypoglycemia or lower blood lipid level, which may not be simply a manifestation of insulin resistance. Given this, several possible explanations may account for this counterintuitive relationship between the TyG index and DF. Firstly, hypoglycemia induced stress response may result in endothelial dysfunction and delayed wound healing [37, 38], thus contributing to DF. Secondly, acute hypoglycemia may activate the systematic inflammation and coagulation pathways [39], thus increasing the risk of DF occurrence. Thirdly, many factors such as malnutrition, infection, and intensive hypoglycemic therapy contributing to hypoglycemia may be risk factors for DF [36].

In this study, compared with participants in the non-DF group, those DF patients had a lower FBG level (7.91±3.9 vs. 9.42±4.65, P < 0.001). However, the DF group showed a higher HbA1c% abnormality rate (> 6.5%) than the non-DF group (75.10% vs. 69.76%, P < 0.001), suggesting a poor glucose management in the long run in DF patients. We speculated that patients with DF had adopted irregular short-term hypoglycemic therapy when they were aware of their poor blood sugar control. The use of insulin and glycosidase inhibitors were increased in the DF group, while insulin secretagogues and biguanides were not. After adjusting for multiple factors, the use of insulin secretagogues was a protective factor for DF (OR 0.61, 95% CI 0.48, 0.78; P < 0.0001). However, the use of biguanides did not show a protective effect (OR 1.01, 95% CI 0.83, 1.23; P = 0.9481). We have reason to believe that the neglect use of insulin secretagogues and biguanides may also contribute to increased risk of DF.

The relationship between DF and serum lipid levels has been previously reported in the literature. The Chinese Diabetes Society suggested that diabetes patients with hyperlipidemia should be instructed to adopt comprehensive treatment, including lifestyle modification and use of statins [40]. Pasto et al. [41] reported that TG was a risk factors for the development of DF ulcers (DFUs) in Albanian patients with T2DM. The mortality rate of vascular disease decreased by 13% for every 39 mg/dl drop in serum LDL-C level among diabetes patients [42]. However, in our study, the DF group had a more favorable lipid profile, including TC, TG and LDL, accompanied by a lower use of fenofibrate. Therefore, the use of fenofibrate on DF was explored and our results showed that the use of fenofibrate decreased the risk of DF by 71% (OR 0.29, 95% CI 0.14–0.59; P < 0.001) when accounting for multiple confounding factors. Treatment with fenofibrate reducing the incidence of DF amputations has been previously reported [43]. Further research is still needed to confirm the effect of fenofibrate on the occurrence of DF. In addition, we speculated that fenofibrate can increase plasma HDL cholesterol levels in diabetes patients, which resulted in a higher HDL-C level in the non-DF group (1.17±0.33) than that in the DF group (1.11±0.31, P < 0.001) in our study. It was in line with a previous meta-analysis, which reported that decreased HDL-C level was associated with the DF occurrence [44].

In our study, DF patients were more likely to be male and elderly, have a longer course of diabetes, have a lower BMI, and smoke and drink alcohol. This was consistent with a previous report that male sex, smoking status and duration of diabetes were risk factors for the occurrence of DFUs [45], and smoking was a hazard factor for DF amputation [46]. We found that DF patients had a higher SBP and an increased PP. This was in line with early reports that poor management of blood pressure was a risk factor for DF infection [6] and postponed wound healing [47]. A cohort study suggested that elevated PP was an independent predictor of foot ulcers in diabetes patients [48], in which the highest quartile of PP had a 2.39-fold (95% CI 1.14–5.02) risk of foot ulcers after adjusting for age, sex, duration of diabetes, SBP, and Charlson comorbidity score.

The major strengths of this study were the large sample size and the adjustment for multifaceted indicators. However, several limitations should be acknowledged: 1) It was a cross-sectional study, which limited the certainty of causality. Due to this limitation, there may be a potential reverse causality, in which DF presence led to abnormal metabolism of blood lipids and glucose. Therefore, prospective cohort studies are warranted to assess the sequence of these associations. 2) This study was based on Chinese diabetes inpatients, which may prevent our results from being generalized to other ethnic and community populations. 3) Potential confounding factors, such as walking habits, shoes and socks, diet and joint deformities, were neglected. 4) The data on smoking were from medical history inquiries, and the cigarette number and duration of smoking cessation could not be obtained. Therefore, only smoking status was included in multiple analyses. In addition, there was also bias in self-reported alcohol consumption. 5) Because of the fewer number of people with DF, the level of DF was not classified.

## Conclusion

In conclusion, the TyG index was inversely and dose-dependently related to the risk of DF in diabetes patients, indicating that elevated TyG index was a protective factor for DF. In addition, the TyG index may be useful for the identification of DF presence in diabetes patients. Future cohort studies and mechanistic studies are therefore warranted to confirm our finding and to explore the detailed pathological mechanism involved in this process.

## Supporting information

S1 Table The association between TyG index and DF in subgroup analysis. TyG, triglyceride glucose index; DF, diabetic foot.
(DOCX)

S2 Table The association between TyG index and DF inT2DM. TyG, triglyceride glucose index; DF, diabetic foot.
(DOCX)

S3 Table The association between TyG index and DF in diabetes with HOMA-IR (n = 4583). HOMA-IR, the homeostasis model assessment of insulin resistance; TyG, triglyceride glucose index; DF, diabetic foot.
(DOCX)

S1 Fig The flow chart of the cross-sectional study. (TIF)

S2 Fig Receiver-operating characteristic analysis for identification of patients with a risk of DF by excluding T1DM. (A)overall population;(B) male; (C) female. DF, diabetic foot.
(TIF)

## References

[1] IDF diabetes atlas 2021. tenth ed 2021 Updated https://diabetesatlasorg [Accessed 3 August 2023]. Published 2021.

[2] Kumar A, Gangwar R, Ahmad Zargar A, Kumar R, Sharma A. Prevalence of diabetes in India: A review of IDF Diabetes Atlas 10th edition. Current diabetes reviews. 2023. Epub 2023/04/19. doi: 10.2174/1573399819666230413094200 . 37069712

[3] Glovaci D, Fan W, Wong ND. Epidemiology of Diabetes Mellitus and Cardiovascular Disease. Current cardiology reports. 2019;21(4):21. Epub 2019/03/05. doi: 10.1007/s11886-019-1107-y . 30828746

[4] Benjamin EJ, Virani SS, Callaway CW, Chamberlain AM, Chang AR, Cheng S, et al. Heart Disease and Stroke Statistics-2018 Update: A Report From the American Heart Association. Circulation. 2018;137(12):e67–e492. Epub 2018/02/02. doi: 10.1161/CIR.0000000000000558 . 29386200

[5] Association AD. Microvascular Complications and Foot Care: Standards of Medical Care in Diabetes-2021. Diabetes care. 2021;44(Suppl 1):S151–s67. Epub 2020/12/11. doi: 10.2337/dc21-S011 . 33298422

[6] Zhang P, Lu J, Jing Y, Tang S, Zhu D, Bi Y. Global epidemiology of diabetic foot ulceration: a systematic review and meta-analysis. Annals of medicine. 2017;49(2):106–16. 27585063 10.1080/07853890.2016.1231932

[7] Singh N, Armstrong DG, Lipsky BA. Preventing foot ulcers in patients with diabetes. Jama. 2005;293(2):217–28. doi: 10.1001/jama.293.2.217 15644549

[8] Horswell RL, Birke JA, Patout CA Jr. A staged management diabetes foot program versus standard care: a 1-year cost and utilization comparison in a state public hospital system. Archives of physical medicine and rehabilitation. 2003;84(12):1743–6. Epub 2003/12/12. doi: 10.1016/s0003-9993(03)00477-5 . 14669177

[9] Mishra SC, Chhatbar KC, Kashikar A, Mehndiratta A. Diabetic foot. BMJ (Clinical research ed). 2017;359. 10.1136/bmj.j5064 PMC5688746 29146579

[10] Lavery LA, Hunt NA, LaFontaine J, Baxter CL, Ndip A, Boulton AJ. Diabetic foot prevention: a neglected opportunity in high-risk patients. Diabetes care. 2010;33(7):1460–2. doi: 10.2337/dc10-0310 20424223 PMC2890341

[11] Boyko EJ, Ahroni JH, Stensel V, Forsberg RC, Davignon DR, Smith DG. A prospective study of risk factors for diabetic foot ulcer. The Seattle Diabetic Foot Study. Diabetes care. 1999;22(7):1036–42. doi: 10.2337/diacare.22.7.1036 10388963

[12] Randrianarisoa E, Lehn-Stefan A, Hieronimus A, Wagner R, Maucher J, Rittig K, et al. Reduced insulin clearance is linked to subclinical atherosclerosis in individuals at risk for type 2 diabetes mellitus. Scientific reports. 2020;10(1):22453. Epub 2021/01/02. doi: 10.1038/s41598-020-80581-x ; PubMed Central PMCID: PMC7775444. 33384433 PMC7775444

[13] DeFronzo RA, Tobin JD, Andres R. Glucose clamp technique: a method for quantifying insulin secretion and resistance. American Journal of Physiology-Endocrinology And Metabolism. 1979;237(3):E214. doi: 10.1152/ajpendo.1979.237.3.E214 382871

[14] Huang R, Cheng Z, Jin X, Yu X, Yu J, Guo Y, et al. Usefulness of four surrogate indexes of insulin resistance in middle-aged population in Hefei, China. Annals of medicine. 2022;54(1):622–32. doi: 10.1080/07853890.2022.2039956 35175162 PMC8856080

[15] Matthews DR, Hosker JP, Rudenski AS, Naylor BA, Treacher DF, Turner RC. Homeostasis model assessment: insulin resistance and beta-cell function from fasting plasma glucose and insulin concentrations in man. Diabetologia. 1985;28(7):412–9. Epub 1985/07/01. doi: 10.1007/BF00280883 . 3899825

[16] Koufakis T, Karras SN, Zebekakis P, Ajjan R, Kotsa K. Should the last be first? Questions and dilemmas regarding early short-term insulin treatment in Type 2 Diabetes Mellitus. Expert opinion on biological therapy. 2018;18(11):1113–21. Epub 2018/09/25. doi: 10.1080/14712598.2018.1526278 . 30244600

[17] Soonthornpun S, Setasuban W, Thamprasit A, Chayanunnukul W, Rattarasarn C, Geater A. Novel insulin sensitivity index derived from oral glucose tolerance test. The Journal of clinical endocrinology and metabolism. 2003;88(3):1019–23. Epub 2003/03/12. doi: 10.1210/jc.2002-021127 . 12629079

[18] Du T, Yuan G, Zhang M, Zhou X, Sun X, Yu X. Clinical usefulness of lipid ratios, visceral adiposity indicators, and the triglycerides and glucose index as risk markers of insulin resistance. Cardiovascular diabetology. 2014;13(1):1–10. doi: 10.1186/s12933-014-0146-3 25326814 PMC4209231

[19] Park K, Ahn CW, Lee SB, Kang S, Nam JS, Lee BK, et al. Elevated TyG Index Predicts Progression of Coronary Artery Calcification. Diabetes care. 2019;42(8):1569–73. Epub 2019/06/12. doi: 10.2337/dc18-1920 . 31182490

[20] Taniguchi A, Nakai Y, Sakai M, Yoshii S, Hamanaka D, Hatae Y, et al. Relationship of regional adiposity to insulin resistance and serum triglyceride levels in nonobese Japanese type 2 diabetic patients. Metabolism: clinical and experimental. 2002;51(5):544–8. Epub 2002/04/30. doi: 10.1053/meta.2002.31984 . 11979383

[21] Taniguchi A, Fukushima M, Sakai M, Miwa K, Makita T, Nagata I, et al. Remnant-like particle cholesterol, triglycerides, and insulin resistance in nonobese Japanese type 2 diabetic patients. Diabetes care. 2000;23(12):1766–9. doi: 10.2337/diacare.23.12.1766 11128349

[22] Tuttolomondo A, Del Cuore A, La Malfa A, Casuccio A, Daidone M, Maida CD, et al. Assessment of heart rate variability (HRV) in subjects with type 2 diabetes mellitus with and without diabetic foot: correlations with endothelial dysfunction indices and markers of adipo-inflammatory dysfunction. Cardiovascular diabetology. 2021;20(1):1–12. 34261479 10.1186/s12933-021-01337-z PMC8281716

[23] Boyko EJ, Ahroni JH, Cohen V, Nelson KM, Heagerty PJ. Prediction of diabetic foot ulcer occurrence using commonly available clinical information: the Seattle Diabetic Foot Study. Diabetes care. 2006;29(6):1202–7. doi: 10.2337/dc05-2031 16731996

[24] Zhao S, Yu S, Chi C, Fan X, Tang J, Ji H, et al. Association between macro-and microvascular damage and the triglyceride glucose index in community-dwelling elderly individuals: the Northern Shanghai Study. Cardiovascular diabetology. 2019;18(1):1–8. 31345238 10.1186/s12933-019-0898-x PMC6657056

[25] Lai JS, Colega MT, Godfrey KM, Tan KH, Yap F, Chong YS, et al. Changes in Diet Quality from Pregnancy to 6 Years Postpregnancy and Associations with Cardiometabolic Risk Markers. Nutrients. 2023;15(8). Epub 2023/04/28. doi: 10.3390/nu15081870 . 37111088 PMC10145322

[26] Hu L, Huang X, You C, Li J, Hong K, Li P, et al. Prevalence of overweight, obesity, abdominal obesity and obesity-related risk factors in southern China. PloS one. 2017;12(9):e0183934. Epub 2017/09/15. doi: 10.1371/journal.pone.0183934 ; PubMed Central PMCID: PMC5598943. 28910301 PMC5598943

[27] Park HM, Lee HS, Lee Y-J, Lee J-H. The triglyceride–glucose index is a more powerful surrogate marker for predicting the prevalence and incidence of type 2 diabetes mellitus than the homeostatic model assessment of insulin resistance. Diabetes research and clinical practice. 2021;180:109042. doi: 10.1016/j.diabres.2021.109042 34506839

[28] Park B, Lee HS, Lee Y-J. Triglyceride glucose (TyG) index as a predictor of incident type 2 diabetes among nonobese adults: a 12-year longitudinal study of the Korean Genome and Epidemiology Study cohort. Translational Research. 2021;228:42–51. doi: 10.1016/j.trsl.2020.08.003 32827706

[29] Lv L, Zhou Y, Chen X, Gong L, Wu J, Luo W, et al. Relationship between the TyG index and diabetic kidney disease in patients with Type-2 diabetes mellitus. Diabetes, Metabolic Syndrome and Obesity: Targets and Therapy. 2021;14:3299. doi: 10.2147/DMSO.S318255 34305401 PMC8296712

[30] Duan S, Zhou M, Lu F, Chen C, Chen S, Geng L, et al. Triglyceride-glucose index is associated with the risk of chronic kidney disease progression in type 2 diabetes. Endocrine. 2023;81(1):77–89. Epub 2023/04/03. doi: 10.1007/s12020-023-03357-z . 37004636

[31] Liu L, Xia R, Song X, Zhang B, He W, Zhou X, et al. Association between the triglyceride–glucose index and diabetic nephropathy in patients with type 2 diabetes: A cross‐sectional study. Journal of diabetes investigation. 2021;12(4):557–65. 33319507 10.1111/jdi.13371 PMC8015837

[32] Pan Y, Zhong S, Zhou K, Tian Z, Chen F, Liu Z, et al. Association between Diabetes Complications and the Triglyceride-Glucose Index in Hospitalized Patients with Type 2 Diabetes. Journal of diabetes research. 2021;2021. doi: 10.1155/2021/8757996 34671683 PMC8523276

[33] Zhou Y, Lu Q, Zhang M, Yang L, Shen X. The U-Shape Relationship between Triglyceride-Glucose Index and the Risk of Diabetic Retinopathy among the US Population. Journal of personalized medicine. 2023;13(3). Epub 2023/03/30. doi: 10.3390/jpm13030495 ; PubMed Central PMCID: PMC10056904. 36983677 PMC10056904

[34] Yao L, Wang X, Zhong Y, Wang Y, Wu J, Geng J, et al. The triglyceride–glucose index is associated with diabetic retinopathy in Chinese patients with type 2 diabetes: a hospital-based, nested, case–control study. Diabetes, Metabolic Syndrome and Obesity: Targets and Therapy. 2021;14:1547. doi: 10.2147/DMSO.S294408 33859485 PMC8043781

[35] Leibowitz G, Raizman E, Brezis M, Glaser B, Raz I, Shapira O. Effects of moderate intensity glycemic control after cardiac surgery. The Annals of thoracic surgery. 2010;90(6):1825–32. Epub 2010/11/26. doi: 10.1016/j.athoracsur.2010.07.063 . 21095319

[36] Peled S, Pollack R, Elishoov O, Haze A, Cahn A. Association of Inpatient Glucose Measurements With Amputations in Patients Hospitalized With Acute Diabetic Foot. The Journal of clinical endocrinology and metabolism. 2019;104(11):5445–52. Epub 2019/06/28. doi: 10.1210/jc.2019-00774 . 31246256

[37] Kiecolt-Glaser JK, Marucha PT, Malarkey WB, Mercado AM, Glaser R. Slowing of wound healing by psychological stress. Lancet (London, England). 1995;346(8984):1194–6. Epub 1995/11/04. doi: 10.1016/s0140-6736(95)92899-5 . 7475659

[38] Christian LM, Graham JE, Padgett DA, Glaser R, Kiecolt-Glaser JK. Stress and wound healing. Neuroimmunomodulation. 2006;13(5–6):337–46. Epub 2007/08/22. doi: 10.1159/000104862 ; PubMed Central PMCID: PMC2792763. 17709956 PMC2792763

[39] Dandona P, Chaudhuri A, Dhindsa S. Proinflammatory and prothrombotic effects of hypoglycemia. Diabetes care. 2010;33(7):1686–7. Epub 2010/07/01. doi: 10.2337/dc10-0503 ; PubMed Central PMCID: PMC2890381. 20587729 PMC2890381

[40] Wang A, Lv G, Cheng X, Ma X, Wang W, Gui J, et al. Guidelines on multidisciplinary approaches for the prevention and management of diabetic foot disease (2020 edition). Burns Trauma. 2020;8:tkaa017. Epub 2020/07/21. doi: 10.1093/burnst/tkaa017 ; PubMed Central PMCID: PMC7336185. 32685563 PMC7336185

[41] Pastore D, Deja-Simoni A, De Stefano A, Pacifici F, Cela E, Infante M, et al. Risk factors for diabetic foot ulcers: an Albanian retrospective study of inpatients with type 2 diabetes. European review for medical and pharmacological sciences. 2022;26(2):558–72. Epub 2022/02/04. doi: 10.26355/eurrev_202201_27883 . 35113432

[42] Kearney PM, Blackwell L, Collins R, Keech A, Simes J, Peto R, et al. Efficacy of cholesterol-lowering therapy in 18,686 people with diabetes in 14 randomised trials of statins: a meta-analysis. Lancet (London, England). 2008;371(9607):117–25. Epub 2008/01/15. doi: 10.1016/S0140-6736(08)60104-X . 18191683

[43] Metelko Z, Brkljacić Crkvencić N. [Prevention of diabetic foot]. Acta medica Croatica: casopis Hravatske akademije medicinskih znanosti. 2013;67 Suppl 1:35–44. Epub 2014/01/01. . 24371974

[44] Pei E, Li J, Lu C, Xu J, Tang T, Ye M, et al. Effects of lipids and lipoproteins on diabetic foot in people with type 2 diabetes mellitus: a meta-analysis. Journal of diabetes and its complications. 2014;28(4):559–64. doi: 10.1016/j.jdiacomp.2014.04.002 24849711

[45] Huang ZH, Li SQ, Kou Y, Huang L, Yu T, Hu A. Risk factors for the recurrence of diabetic foot ulcers among diabetic patients: a meta‐analysis. International Wound Journal. 2019;16(6):1373–82. doi: 10.1111/iwj.13200 31489774 PMC7949075

[46] Liu M, Zhang W, Yan Z, Yuan X. Smoking increases the risk of diabetic foot amputation: A meta-analysis. Experimental and therapeutic medicine. 2018;15(2):1680–5. doi: 10.3892/etm.2017.5538 29434753 PMC5774386

[47] Kee KK, Nair HK, Yuen NP. Risk factor analysis on the healing time and infection rate of diabetic foot ulcers in a referral wound care clinic. Journal of wound care. 2019;28(Sup1):S4–S13. doi: 10.12968/jowc.2019.28.Sup1.S4 30724120

[48] Monami M, Vivarelli M, Desideri CM, Colombi C, Marchionni N, Mannucci E. Pulse pressure and prediction of incident foot ulcers in type 2 diabetes. Diabetes care. 2009;32(5):897–9. Epub 2009/02/07. doi: 10.2337/dc08-1679 ; PubMed Central PMCID: PMC2671124. 19196880 PMC2671124

