# Correction: Counterintuitive relationship between the triglyceride glucose index and diabetic foot in diabetes patients: A cross-sectional study

**DOI:** 10.1371/journal.pone.0336351

**Published:** 2025-11-05

**Authors:** The PLOS One Staff

There are errors in the author affiliations. The correct affiliations are as follows:

Zhaoping Li^1^, Minghao Zhang^1^, Lulu Han^1^, Lili Fu^1^, Yixia Wu^1^, Haiyan Chen^2^, Li Feng^1^

1 Department of Clinical Nutrition, Shandong Provincial Hospital Affiliated to Shandong First Medical University, Jinan, Shandong, China, 2 Department of Geriatric Endocrinology, Shandong Provincial Hospital Affiliated to Shandong First Medical University, Jinan, Shandong, China
