# Supplementary material for: Counterintuitive relationship between the triglyceride glucose index and diabetic foot in diabetes patients: A cross-sectional study
Source: PLoS One. 2023 Nov 3;18(11):e0293872. doi: 10.1371/journal.pone.0293872 (PMC10624312; doi:10.1371/journal.pone.0293872)
Supplement: S1 Table — TyG, triglyceride glucose index; DF, diabetic foot. (DOCX) [file pone.0293872.s001.docx]

**Table S1 The association between TyG index and DF in subgroup analysis**

|  | TyG index, quartile | | | | **P_trend_** | P interaction |
| --- | --- | --- | --- | --- | --- | --- |
|  | Q1 | Q2 | Q3 | Q4 |  |  |
| **gender** |  |  |  |  |  |  |
| male | Ref | 0.79 (0.59, 1.05) | 0.57 (0.41, 0.80) | 0.45 (0.30, 0.70) | <0.001 | 0.4421 |
| female | Ref | 0.66 (0.46, 0.95) | 0.58 (0.39, 0.87) | 0.31 (0.18, 0.53) | <0.001 |  |
| **age** |  |  |  |  |  |  |
| <60 year | Ref | 0.58 (0.39, 0.85) | 0.43 (0.27, 0.66) | 0.35 (0.21, 0.58) | <0.001 | 0.3128 |
| ≥60 year | Ref | 0.85 (0.65, 1.11) | 0.67 (0.49, 0.92) | 0.37 (0.23, 0.59) | <0.001 |  |
| **overweight** |  |  |  |  |  |  |
| BMI<24kg/m^2^ | Ref | 0.70 (0.50, 0.99) | 0.37 (0.24, 0.57) | 0.36 (0.21, 0.64) | <0.001 | 0.1641 |
| BMI≥24kg/m^2^ | Ref | 0.79 (0.59, 1.07) | 0.71 (0.51, 0.99) | 0.43 (0.28, 0.65) | <0.001 |  |

Adjusted for age, sex, smoking, drinking,body mass index, duration of diabetes, pulse pressure, total cholesterol, LDL cholesterol, HDL cholesterol, APOA/APOB, total serum albumin, prealbumin, globulin, hemoglobin,platelets, white blood cell, red blood cell, numbers of neutrophil, glutamic-pyruvic transaminase, creatinine, uric acid, glycosylated hemoglobin, C reactive protein, fenofibrate agents, statin drugs, insulin, insulin secretagogues, bisguanides, glycosidase inhibitors, thiazolidinediones and DPP4 inhibitor except for the stratified variables.
