# Supplementary material for: Counterintuitive relationship between the triglyceride glucose index and diabetic foot in diabetes patients: A cross-sectional study
Source: PLoS One. 2023 Nov 3;18(11):e0293872. doi: 10.1371/journal.pone.0293872 (PMC10624312; doi:10.1371/journal.pone.0293872)
Supplement: S2 Table — TyG, triglyceride glucose index; DF, diabetic foot. (DOCX) [file pone.0293872.s002.docx]

**Table S2 The association between TyG index and DF inT2DM (n=8587)**

| **TyG index** | | continuous | TyG index, quartile | | | | **P_trend_** |
| --- | --- | --- | --- | --- | --- | --- | --- |
|  |  |  | Q1 | Q2 | Q3 | Q4 |  |
| **Odds ratio (95% CI)** | **Model 1** | 0.46 (0.42, 0.51) | Ref | 0.62 (0.51, 0.74) | 0.39 (0.32, 0.48) | 0.19 (0.14, 0.24) | <0.001 |
|  | **Model 2** | 0.55 (0.49, 0.61) | Ref | 0.68 (0.57, 0.82) | 0.47 (0.38, 0.58) | 0.28 (0.22, 0.37) | <0.001 |
|  | **Model 3** | 0.55 (0.49, 0.61) | Ref | 0.68 (0.56, 0.82) | 0.46 (0.37, 0.57) | 0.28 (0.21, 0.37) | <0.001 |
|  | **Model 4** | 0.68 (0.59, 0.79) | Ref | 0.76 (0.61, 0.94) | 0.59 (0.46, 0.77) | 0.41 (0.29, 0.56) | <0.001 |

Model 1: unadjusted

Model 2: adjusted for age and sex;

Model 3: further adjusted for smoking and drinking;

Model 4: further adjusted for body mass index, duration of diabetes, pulse pressure, total cholesterol, LDL cholesterol, HDL cholesterol, APOA/APOB, total serum albumin, prealbumin, globulin, hemoglobin,platelets, white blood cell, red blood cell, numbers of neutrophil, glutamic-pyruvic transaminase, creatinine, uric acid, glycosylated hemoglobin, C reactive protein, fenofibrate agents, statin drugs, insulin, insulin secretagogues, bisguanides, glycosidase inhibitors, thiazolidinediones and DPP4 inhibitor.
