# Supplementary material for: Counterintuitive relationship between the triglyceride glucose index and diabetic foot in diabetes patients: A cross-sectional study
Source: PLoS One. 2023 Nov 3;18(11):e0293872. doi: 10.1371/journal.pone.0293872 (PMC10624312; doi:10.1371/journal.pone.0293872)
Supplement: S3 Table — HOMA-IR, the homeostasis model assessment of insulin resistance; TyG, triglyceride glucose index; DF, diabetic foot. (DOCX) [file pone.0293872.s003.docx]

**Table S3 The association between TyG index and DF in diabetes with HOMA-IR (n=4583)**

| **TyG index** | | continuous | TyG index, quartile | | | | **P_trend_** |
| --- | --- | --- | --- | --- | --- | --- | --- |
|  |  |  | Q1 | Q2 | Q3 | Q4 |  |
| **Odds ratio (95% CI)** | **Model 1** | 0.49 (0.42, 0.58) | Ref | 0.60 (0.45, 0.80) | 0.41 (0.29, 0.56) | 0.20 (0.14, 0.31) | <0.001 |
|  | **Model 2** | 0.58 (0.49, 0.69) | Ref | 0.67 (0.50, 0.90) | 0.50 (0.36, 0.69) | 0.31 (0.21, 0.47) | <0.001 |
|  | **Model 3** | 0.57 (0.48, 0.68) | Ref | 0.67 (0.50, 0.90) | 0.49 (0.36, 0.68) | 0.31 (0.20, 0.47) | <0.001 |
|  | **Model 4** | 0.69 (0.55, 0.88) | Ref | 0.73 (0.52, 1.03) | 0.65 (0.43, 0.97) | 0.38 (0.22, 0.63) | <0.001 |

Model 1: unadjusted

Model 2: adjusted for age and sex;

Model 3: further adjusted for smoking and drinking;

Model 4: further adjusted for body mass index, duration of diabetes, pulse pressure, total cholesterol, LDL cholesterol, HDL cholesterol, APOA/APOB, total serum albumin, prealbumin, globulin, hemoglobin, platelets, white blood cell, red blood cell, numbers of neutrophil, glutamic-pyruvic transaminase, creatinine, uric acid, glycosylated hemoglobin, C reactive protein, fenofibrate agents, statin drugs, insulin, insulin secretagogues, bisguanides, glycosidase inhibitors, thiazolidinediones and DPP4 inhibitor.
