# Supplementary figures and images for: Counterintuitive relationship between the triglyceride glucose index and diabetic foot in diabetes patients: A cross-sectional study
Source: PLoS One. 2023 Nov 3;18(11):e0293872. doi: 10.1371/journal.pone.0293872 (PMC10624312; doi:10.1371/journal.pone.0293872)

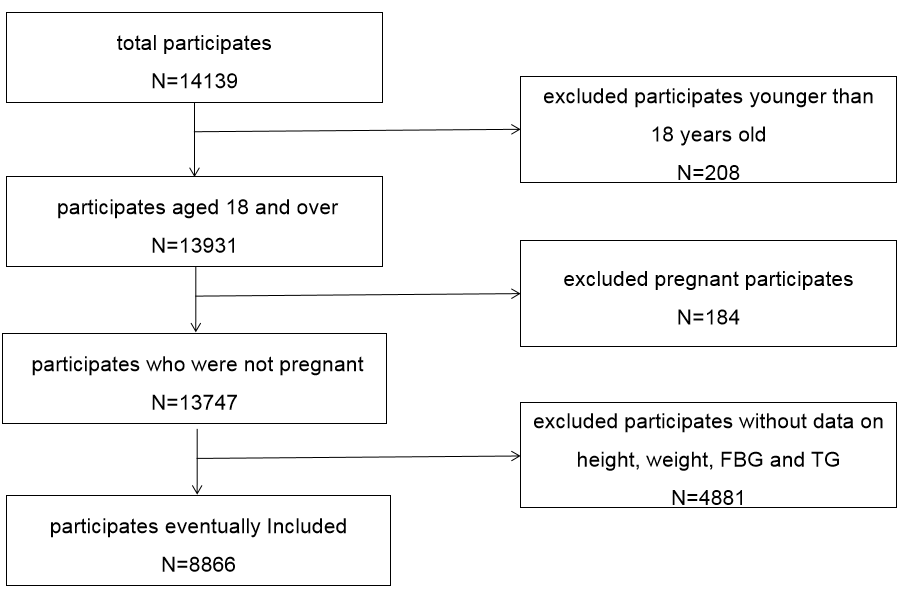

Supplement: S1 Fig — (TIF) [file pone.0293872.s004.tif]

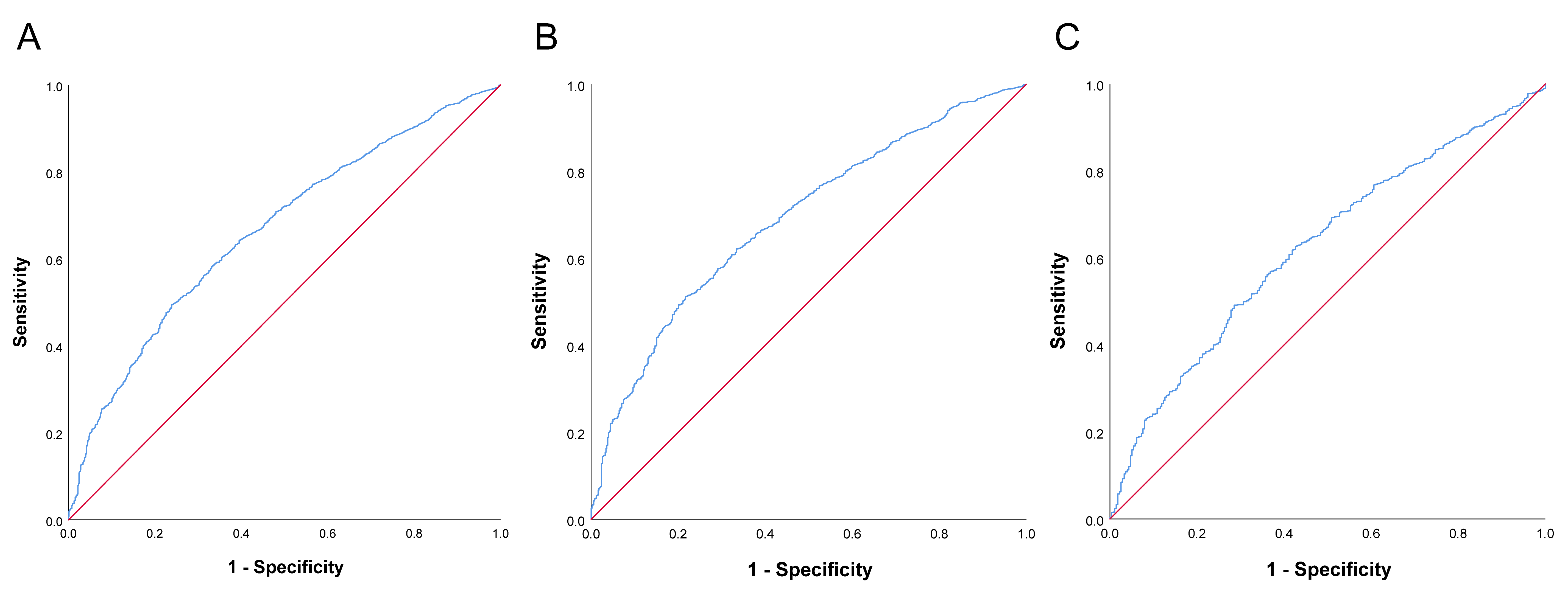

Supplement: S2 Fig — (A)overall population;(B) male; (C) female. DF, diabetic foot. (TIF) [file pone.0293872.s005.tif]
